# Case managers’ reflections of a brief case management intervention in Canada

**DOI:** 10.3389/fpsyt.2023.1151904

**Published:** 2023-06-28

**Authors:** Andrea Duncan, Maritt Kirst, Katie N. Dainty, Walter P. Wodchis, Vicky Stergiopoulos

**Affiliations:** ^1^Department of Occupational Science and Occupational Therapy, University of Toronto, Toronto, ON, Canada; ^2^Community Psychology, Wilfrid Laurier University, Waterloo, ON, Canada; ^3^Institute of Health Policy, Management and Evaluation, University of Toronto, Toronto, ON, Canada; ^4^Center for Addiction and Mental Health, Toronto, ON, Canada

**Keywords:** community, mental health, case management, brief case management, complex intervention

## Abstract

**Introduction:**

As demand for intensive case management services continues to outpace supply, community mental health agencies in Toronto, Ontario, introduced Short-Term Case Management (STCM).

**Objective:**

This study sought to explore case managers’ perspectives and experiences with this new service delivery model.

**Methods:**

Focus groups were conducted with twenty-one case managers, and transcripts analyzed using thematic analysis.

**Results:**

Emerging themes suggest that despite embracing a recovery approach, case managers expressed mixed views on the acceptability and appropriateness of this service delivery model as an intervention.

**Conclusion:**

The ideal population for this intervention are adults with mental health issues in need of system navigation, and those motivated to address their goals. Further research is needed to establish fidelity criteria.

## 1. Introduction

The goal of community-based mental health services is to “promote stability, recovery and opportunities for employment and social reintegration” (1). A commonly used community mental health intervention for people with serious mental illness is Intensive Case Management (ICM) (2). Within ICM, a client works directly with a case manager to improve their health, develop coping skills, strengthen community connections, and increase their quality of life (3). Case managers typically meet with clients in the community on a weekly basis (4) and focus on system navigation, psychoeducation and skill development (5). A recovery-based philosophy is often embraced in ICM, with the goal of keeping it flexible, individualized and customizable in its delivery. Using this approach within an intervention is about supporting clients to improve quality of life, personal satisfaction and increase hope and resiliency (6). At the same time, case managers have various styles, training and levels of supervision; therefore, the delivery of ICM can be highly variable. Despite its common use in practice, the evidence on the effectiveness of ICM to improve clients’ quality of life is of low to moderate quality (2).

In Ontario, the demand for ICM services has risen, resulting in an increase in waiting times for services. To address the growing waitlist, in 2016, two community mental health agencies conceptualized, designed and introduced an intervention called Short-Term Case Management (STCM). STCM, like ICM, is a community-based intervention where a client works with a case manager to address personalized goals and unmet needs. Both intervention approaches typically have case managers meet with clients once a week for approximately an hour, however, the distinguishing feature of STCM is that it is time limited and restricted to up to 3 months of services. This is in contrast to ICM services which may be ongoing for many years (7).

The concept of STCM was inspired by Critical Time Intervention (CTI) (8). CTI is an evidence informed intervention focused on supporting individuals in crisis or in transition from institutional to community settings. CTI is well established in the literature and has been shown to be effective with a number of populations experiencing mental health problems and illnesses; such as men experiencing homelessness (9), children in high risk families (10) and women who have a history of domestic abuse (11). While CTI informed the early conceptualization of STCM, it was created as an approach intended to address the needs of service users on the waiting list.

While there is evidence to support that short-term therapeutic relationships can have an impact on clients’ health and well-being (12, 13), few evaluations of brief case management interventions have been conducted to date. One preliminary evaluation did not demonstrate positive outcomes for frequent users of Emergency Departments. Specifically, Stergiopoulos et al. (14) found that the provision of brief case management was not statistically associated with reduced emergency department usage. In addition, the study found no significant improvements among clients who received brief case management services, compared to a control group of participants on health, recovery or quality of life outcomes. The authors highlighted the challenges associated with evaluating community-based interventions with highly variable implementation and fidelity to the care model (14).

Given the novelty of the STCM model in Toronto, Ontario, and questions about its effectiveness, the unique perspectives and experiences of front-line case managers can highlight insights and lessons learned to guide further development. Unique perspectives of service users (15) and the evaluation of the model itself (16) have been published elsewhere. While STCM was inspired by an evidence informed service delivery model, local agencies shared that they did not adhere to the CTI protocol. Additionally, variations in the local service delivery context, clients’ needs, service providers’ skills and program resources are key components that can influence implementation and success of any case management service model. This study sought to explore case managers’ perspectives on the short-term case management delivery model and understand their experiences with this new time constricted approach.

## 2. Methods

### 2.1. Data collection

We employed a qualitative descriptive design in this study (17). Focus groups were used to collect data from case managers from the two service agencies who developed and offered STCM. Twenty-one case managers participated in one of three focus groups held in November 2019.Case managers who had experience with providing STCM services for 6 months or longer were deemed eligible. Participants were recruited via email directly from their managers; however, participation in the study was optional and consent to participate or not was not communicated back to managers. This study received REB approval from the University of Toronto on September 19th, 2019, with protocol # 00038074. Focus group discussions, conducted in November 2019, were guided by a semi-structured interview guide and facilitated by one author. The interview guide was developed in consultation with case managers and the senior managers with participating organizations. See Appendix A. A work study student was present for all three focus groups to capture field notes of case managers interactions. Additional fieldnotes were also written after each focus group to capture facilitator’s thoughts and reflections in advance of data analysis.

### 2.2. Data analysis

All focus groups were audio recorded and transcribed verbatim by an external transcriptionist. All transcribed data were then managed using NVivo (18) software. With no a priori theory, analysis began with the lead author reading and reviewing the transcripts of all focus groups and reflectively reviewing field notes. This led to the creation of a preliminary list of themes which were captured in a code book within NVivo which was used for data coding rigor. Two reviewers then deductively coded one focus group transcript independently. Codes were compared and discussed until consensus was reached. The two subsequent focus groups were coded by one author. Next, the data within each code was reviewed as we sought to analyze and understand thematic patterns. The research team discussed and refined the thematic analysis as we formulated our final conclusions (19).

## 3. Results

Emerging themes suggest that case managers, despite embracing the recovery philosophy inherent in the model, had mixed experiences with delivering the STCM intervention. They highlight that short term case management may be best suited for adults with mental health issues in need of system navigation and those motivated to address their personal goals. They also described that the absence of feedback loops on individual and program level outcomes was a barrier to continuing improvement of the model and its practice.

### 3.1. Embracing recovery as the underlying program philosophy

The most consistent theme arising within and across the focus groups was that case managers embrace the recovery approach and philosophy within STCM. Many case managers talked about “meeting the client where they are at” and aiming to find ways to make small improvements to the clients’ quality of life as key priorities. They described a strong focus on client driven care plans, and on instilling hope, as this case manager identified.

*Hope is another {outcome}. I think when you have a short time, you really want to instill hope in people that it will get better.* Female Case Manager, Focus Group #1.

While most supported the model’s recovery philosophy, a few case managers described challenges with the approach within a time restricted intervention. Specifically, they described challenges arising for those with more complex needs or not yet ready to identify or discuss their personal goals.

*I think we all sort of work with the recovery model in the back of our minds. Is that our service should be client-directed, and it should be looking at, you know, what do they want to achieve, how do they want to improve their quality of life? But when we come to short term case management, that almost kind of goes out the window.* Male Case Manager, Focus Group #2.

### 3.2. Polarized views of the STCM model

Analysis of case managers’ reflections exposed variable levels of engagement and commitment to the STCM service delivery model. Some case managers expressed an appreciation for how setting a time restriction can help some clients focus on their goals and avoid dependency on the program. However, others described discomfort with clients with enduring needs for only 3 months. They raised ethical concerns about discharging clients to few or no supports, and little progress.

*I do not know, I do not personally agree with short term case management. It’s just that I’ve done all my best work working with people on a long-term setting. And just on our team, the expectation is 3 months … I just think you need more time to develop that relationship and really start to get things completed. Because even if you do the referral, they might not go to it or they might not like it. It requires weeks of follow … And things come up where they need further help with paperwork. So, I just feel like having a consistent person there to help them, and someone that they know and that they trust is important.* Female Case Manager, Focus Group #2.

*I think for myself; I’ve had some positive experiences through short term … there’s a shift in the way I present case management and the language that I use. And realizing that really just having to present what would be the most meaningful to you right now to improve your quality of life right now.* Female Case Manager, Focus Group #2.

These two quotes from two different case managers within the same focus group represent the divided views of case managers on the model.

### 3.3. Identifying the ideal population for STCM

There was no consensus within or across focus groups on specific mental health conditions that might best be suited for STCM. However, case managers concurred that clients with substance use issues may not be best suited for STCM, as their needs may not be successfully addressed within a three-month period of time.

*And you know, in that case, like are we perpetuating like for this person that everyone else has given up on them, and we are too? Because we are not able to put in that extra bit of time to work on helping them change those behaviours. Like you know, working on the stages of change with that person, in particular people with addictions. And we are just kind of giving up on them… That’s something I think about as well. You know, are we just kind of perpetuating that same kind of thing for those people? It’s difficult. It’s difficult to navigate.* Female Case Manager, Focus Group #1.

Case managers highlighted that STCM is a broker case management intervention, in that the case managers’ role is to connect a client with other services, offering system navigation. In this context, they described that clients who did well with STCM were those in urgent need of being connected to resources in the community, irrespective of level of need or diagnosis.

*I actually felt like it was fantastic for people who had little or no experience with the mental health system. I think most of the clients I met with were very appreciative of the fact that, “Oh, okay … I did not know about these resources. I did not know where to go for this or for housing.” I think it was very good for them generally.* Female Case Manager, Focus Group #2.

Furthermore, there was consensus among case managers that the populations most likely to benefit for STCM are those that already have some support systems in place and those motivated and ready to work on their goals.

*So, someone who comes to you with nothing in place, that might be harder to achieve than someone who has some existing supports already.* Male Case Manager, Focus Group #1.

### 3.4. Measuring needs and outcomes for program improvement

Case managers described collecting data on client levels of need through the Ontario Common Assessment of Need (OCAN), as part of the assessment process and again at the time of discharge. Although some case managers found the tool useful in identifying clients’ needs, most found the tool unhelpful in the context of STCM, highlighting it is the same tool used for long term ICM. Most case managers identified instead the need for more focused assessments and planning tools, tailored to what is achievable within a short-term intervention.

*I think one of the process challenges for me is we are using the same tools. So the same OCAN, the same safety plan, the same…for long term that we are using for short term. That really needs to change. If we are moving towards short term, we have to have assessments that fit. … So what is the priority? So it’s very different language, it’s very different process.* Male Case Manager, Focus Group #3.

Case managers also identified that although OCAN data was completed at the time of discharge, it was often not shared with case managers, nor was there an opportunity to reflect on the success of the intervention from both the case manager and the client perspective. In addition to missed opportunities to reflect on individual client outcomes, case managers identified that there was no feedback loop of program level data to inform improvement efforts.

*I think we need better data around how effective we are being and whether or not we are doing the right things. I do not mind being accountable. I just want the information.* Male Case Manager, Focus Group #3.

### 3.5. Limitations of the service delivery context

Lastly, challenges with delivering STCM within the local community mental health system were often described. These challenges reflected difficulties in referring suitable clients to STCM, and in achieving continuity of care for those with more complex needs.

Starting from the point of referral, case managers identified that privacy laws and policies often prevent the transfer of sufficient information across agencies, limiting their ability to engage with a client appropriately.

*I think another element is we used to get a lot of professional referrals. So we used to have a lot of information, right. So clinically meeting the client for the first time, we were halfway there. Now we are getting self-referrals or we are getting referrals from a neighbour or from a housing worker. And so we are actually starting off with zero information clinically. And clients may not disclose what they need to. So you are like towards the end going, “Oh my gosh, that diagnosis is so not right,” or I just found out that they are in an abusive relationship, they are being evicted, they have hoarding. So it’s very challenging.* Male Case Manager, Focus Group #3.

They also described receiving referrals they deemed inappropriate, reflecting lack of understanding by referring agencies of what STCM can offer.

*Social workers in hospital, their discharge plan was, “Oh, rapid response case management, they’ll take care of it.” Housing providers were saying, “We do not know what to do with them. We’re going to evict this client. Case management.” Like we became the catch-all for we are going to fix everything. And that was on a systemic level, not from the client perspective. That was from actual referral … when people were being prepared to be discharged. I’m not saying these clients might not have been appropriate, but this is not the answer for everything.* Female Case Manager, Focus Group #2.

Finally, case managers described the lack of availability of housing and long-term mental health supports as a significant system challenge, impacting their access to services within a broker case management model.

*And there’s nothing to refer them to. The odd time I would take someone actually to a housing help centre, and it would be a complete waste of time … There’s nothing for people. So if I have a 25, 26 year old male or female, not a lot of experience in the system, you know, sleeping rough or kind of somewhat vicariously, or their parents want them out, where do I house them? … So yeah, I think the lack of like real systems and a support system in the Greater Toronto Area to refer people to was the biggest challenge I faced. Because I can do a great job, I can be well-meaning … but if you do not have $1,200 for rent, and you still have to eat, what do you do?* Male, Case Manager, Focus Group #3.

## 4. Discussion

This study explored case manager perspectives on and experiences with short term case management, an intervention launched in Toronto, Ontario to address the growing waiting lists for Intensive Case Management Services. Despite embracing the recovery approach and philosophy of this brokered case management model, case managers expressed mixed views on the acceptability and appropriateness of short-term case management. Some case managers appreciated that the short-term model prevented dependency on a case manager, empowering clients to work on their recovery goals. Other case managers described difficulty making meaningful connections with clients in a short-term time frame. Of note, some case managers participated in the design and implementation of the intervention, while others did not. It might be hypothesized that those engaged in the design and implementation of the model may have had more positive experiences.

Case managers described using common assessment planning and outcome evaluation tools at intake and discharge (e.g., Adapted version of the OCAN). They also highlighted, despite responsibilities to collect client data, neither individual nor program level data was shared with them as part of a continuous improvement process. Feedback on performance, impact and outcomes has increasingly been recognized as an essential component of improving performance in healthcare, and many researchers are recommending the implementation of feedback loops (20). The presence of audit and feedback loops, used in health care to provide professionals with feedback on performance, has been shown to improve provider performance and patient outcomes (21).

Study findings therefore highlight the need for community mental health agencies to explore how best to establish mechanisms of supervision and feedback to front-line case managers, including sharing of and reflection on outcomes, routinely collected by community mental health agencies. The Critical Time Intervention model, which inspired STCM, has a robust protocol that includes details of supervision and skill building of case managers (11). If brief case management interventions are to be used, closer attention to evidence informed approaches is recommended.

In describing the ideal population for STCM, case managers concurred that the health and social needs of clients are complex, and that complex community-based interventions are needed to support them. The peer-reviewed literature offers numerous best practice examples and process guidelines regarding how complex interventions should be designed and evaluated (22–24). More specifically, O’Cathain et al. (23) offer a framework for complex intervention development that includes steps such as involving stakeholders from various backgrounds, reviewing evidence and developing structured data collection and evaluation approaches. In our communication with the two community mental health agencies in planning this study and during data collection with the case managers, it did not appear that a robust process to design STCM was embraced. While STCM was communicated as based on CTI and brief counselling approaches, it became apparent during data collection that an evidence informed service delivery model was not implemented. This suggests that there was lack of rigor in implementation. It is recommended that new approaches to care in community mental health leverage best practices for complex intervention development and evaluation and engage all key stakeholders in this process.

A reflective question that arose during this study is *what is the right length of service time for someone who is experiencing a serious mental illness in the community?* While our study findings suggest that some positive outcomes can be seen within a short three-month period for some clients, STCM cannot address all clients’ goals and needs in this time frame. On the one hand, a system that does not retain clients in services for many years may be desirable, as it avoids dependency on services, promoting instead empowerment and illness self-management. The recovery approach to services gives people control over and responsibility over their own lives (25), allowing for resiliency building as clients are empowered to do things for themselves. In that context, it may be helpful that the intervention supports clients in the early stages of their recovery journey and connects them as needed to long term supports. With finite resources available in our health and social system, avoiding service dependency amongst our service user populations is an important goal. Conversely, the ethical challenges of prematurely discharging clients after 3 months of service delivery cannot be ignored. This is an important issue and one that would require further understanding and investigation. Designing community health programs that meet individual needs, but also maintain fiscal accountability is a wicked problem (26) and one that is not easy to solve.

### 4.1. Limitations

This study focused on an intervention developed by two community mental health agencies in Toronto, Ontario, therefore our study has issues of external validity. How this model works within an urban and well-resourced environment, may be different from its relevance and impact in an alternate service delivery context. Other geographies may have more or less resources, may present populations with different problems and illnesses and there may be different levels of understanding and application the short-term model by case managers. Additionally, twenty-one case managers presents as a small sample size and a larger sample may have found different results. Even with these limitations, the challenges in our setting are shared with many other jurisdictions facing growing demand for case management services and highlight important considerations for future development.

## 5. Conclusion

Short term case management may have an important role to play in addressing the growing waiting lists for case management services, however more work is needed to define the model, target outcomes, and ideal population to service. Additionally, ensuring case managers are committed to the service deliver model is essential. In the case of this brief case management intervention, mixed views on this model of care were expressed by the case managers. Strong stakeholder engagement is needed when designing complex interventions, to ensure program acceptability and appropriateness and to facilitate program success. Future research should establish fidelity criteria for short term case management and evaluate its success. This evolving evidence will support case managers to more fully embrace this service delivery model.

## Data availability statement

The original contributions presented in the study are included in the article/Supplementary materials, further inquiries can be directed to the corresponding author.

## Ethics statement

The studies involving human participants were reviewed and approved by this study received REB approval from the University of Toronto on September 19th, 2019, with protocol # 00038074. The patients/participants provided their written informed consent to participate in this study.

## Author contributions

AD, MK, KD, WW, and VS contributed to the study conception and design. Data collection and analysis were performed by AD. The first draft of the manuscript was written by AD and MK, KD, WW, and VS commented on previous versions of the manuscript. All authors contributed to the article and approved the submitted version.

## Conflict of interest

The authors declare that the research was conducted in the absence of any commercial or financial relationships that could be construed as a potential conflict of interest.

## Publisher’s note

All claims expressed in this article are solely those of the authors and do not necessarily represent those of their affiliated organizations, or those of the publisher, the editors and the reviewers. Any product that may be evaluated in this article, or claim that may be made by its manufacturer, is not guaranteed or endorsed by the publisher.
