# Supplementary material for: Case managers’ reflections of a brief case management intervention in Canada
Source: Front Psychiatry. 2023 Jun 28;14:1151904. doi: 10.3389/fpsyt.2023.1151904 (PMC10336220; doi:10.3389/fpsyt.2023.1151904)
Supplement: Supplementary file 1 [file Data_Sheet_1.PDF]

Appendix A – Short Term Case Manager Focus Group Discussion Guide

1. Can you please share with me the process of Short-Term Case Management?
   Probing Questions
   a. What are the key steps in this intervention?
   b. How do you progress along these steps?
   c. Is there one stage that is more challenging than another? Are there ones that are easier?
2. Can you share with me any guiding principles that guide your provision of Short-Term Case Management?
   Probing Questions
   a. How were these guiding principles established?
   b. Can you share with me when it is harder or easier to follow these principles?
   c. When are these principles most effective?
3. What are realistic and achievable outcomes of short-term case management?
   Probing Questions
   a. How do the guiding principles help you achieve success with clients?
   b. What "needs" are you able to address with clients?
   c. What "needs" do you feel do not get addressed with clients?
   d. Why is this?

Now I would like to share some findings with you. We looked at the OCAN data from 2016 – 2018. We looked at service user and service provider assessment of need. We also looked at the initial and discharge findings. (*findings shared in a handout*)

4. What is your initial reaction to this data?
   Probing Questions
   a. Is this information surprising, or is it what you would expect to see?
   b. If it is not what you would expect, why do you think this is?
5. Can you help me further understand what the OCAN data is highlighting about your STCM?
   Probing Questions
   a. What is the key take-away message about the impact of STCM?
   b. What would you like to contextualize to help me further understand this data?
   c. What is missing from this data?
6. Is there anything further you would like to share about the guiding principles or expected outcomes of Short-Term Case Management?

Thank you for your time today.
